# ﻿*Allopachrianigrocatta* sp. nov. from Guangxi, with a key and checklist of Chinese species and additional records of *Allopachria* Zimmermann, 1924 from China (Coleoptera, Dytiscidae, Hydroporinae, Hyphydrini)

**DOI:** 10.3897/zookeys.1219.137179

**Published:** 2024-12-04

**Authors:** Zhuo-yin Jiang, Ya-ru Chen, Li-bo Han, Feng-long Jia, Bei-xin Wang

**Affiliations:** 1 Department of Entomology, College of Plant Protection, Nanjing Agricultural University, Nanjing 210095, China Sun Yat-sen University Guangzhou China; 2 Department of Entomology, College of Life Sciences, Sun Yat-sen University, Guangzhou 510275, China Nanjing Agricultural University Nanjing China

**Keywords:** *Allopachria*, checklist, China, Dytiscidae, fauna, Hydroporinae, identification key, new record, new species, Oriental region, taxonomy

## Abstract

A new species *Allopachrianigrocatta***sp. nov.** from Guangxi, China is described. New records are provided for the following *Allopachria* species: *A.dudgeoni* Wewalka, 2000 and *A.weinbergeri* Wewalka, 2000 from Zhejiang, and *A.yiae* Bian, Guo & Ji, 2013 from Guizhou. Additional records are also given for some other Chinese species. The habitus and male genitalia of the new species are illustrated. An updated key and a checklist of all Chinese species of *Allopachria* are provided.

## ﻿Introduction

*Allopachria* Zimmermann, 1924, are small to extremely small beetles, most of which bear maculae on their elytra. Male specimens of some species have antennomeres, protibiae or protarsomeres modified in various ways (Wewalka 2000). Species of the genus occur mainly in the Oriental region, from Indonesia in the south to southern China and Japan in the north (Wewalka 2010). *Allopachria* typically live by the margin of flowing waters, usually with a sand or stone bottom. The knowledge of *Allopachria* was very poor until Wewalka (2000) made a comprehensive revision of the genus. Then, the studies increased gradually, and the fauna of Chinese *Allopachria* was also supplemented (Bian and Ji 2010; Wewalka 2010; Bian and Ji 2012; Bian et al. 2013; Ji et al. 2014). Up to now, the genus contains 47 species worldwide (Nilsson and Hájek 2024a), of which 29 species occur in China (Nilsson and Hájek 2024b).

In the present study, we describe a new species from Guangxi, China and provide additional records for some Chinese *Allopachria* species. For the first time, an updated key to all known species from China is provided.

## ﻿Materials and methods

Specimens were examined and measured under a Nikon SMZ800N stereomicroscope. Some of the specimens were dissected, and the genitalia were put into 10% KOH at room temperature for 8–10 h, then placed in a drop of glycerol on glass slides for photographing. Photographs of habitus and characters were taken with a Nikon DS-Ri2 mounted on a Nikon SMZ25; layers were captured and aligned in the NIS-Elements software. Photographs of genitalia were taken with a Zeiss AxioCam HRc mounted on a Zeiss AX10 microscope with the Axio Vision SE64 software, then stacked in Helicon Focus (ver. 7.0.2). After being photographed, the genitalia were transferred to a transparent plastic plate in a drop of glycerol and attached to the respective specimen. The images were edited and assembled with Adobe Photoshop CS6.

The following abbreviations were used in the descriptions: TL, total length, measurement of length from clypeal margin to apex of elytra; TL-h, total length minus head length, measurement of length from anterior margin of pronotum to apex of elytra; MW, maximum width of body measured at right angle to TL. The terminology follows Wewalka (2000) and Wewalka (2010), and the style of description of the new species follows Jiang et al. (2022). The terminology to denote the orientation of the genitalia follows Miller and Nilsson (2003). Exact label data are cited for the type material and given in quotation marks. Authors’ additional remarks are provided in square brackets; [p]–preceding data are printed. Separate label lines are indicated by a slash (/), and separate labels by a double slash (//).

The specimens included in this study are deposited in the following collections:

**SYSU**Biological Museum, Sun Yat-sen University, Guangzhou, China.

**ZJCQ** Zhuo-yin Jiang collection, Quzhou, China.

## ﻿Taxonomy

### ﻿ Allopachria nigrocatta

Taxon classificationAnimaliaColeopteraDytiscidae

Jiang & Jia sp. nov.

05D20B42-D185-5309-BB64-17618B07EE22

https://zoobank.org/0D13517E-1D9A-4171-AFB3-CD19D5880446

**Figure 1.**
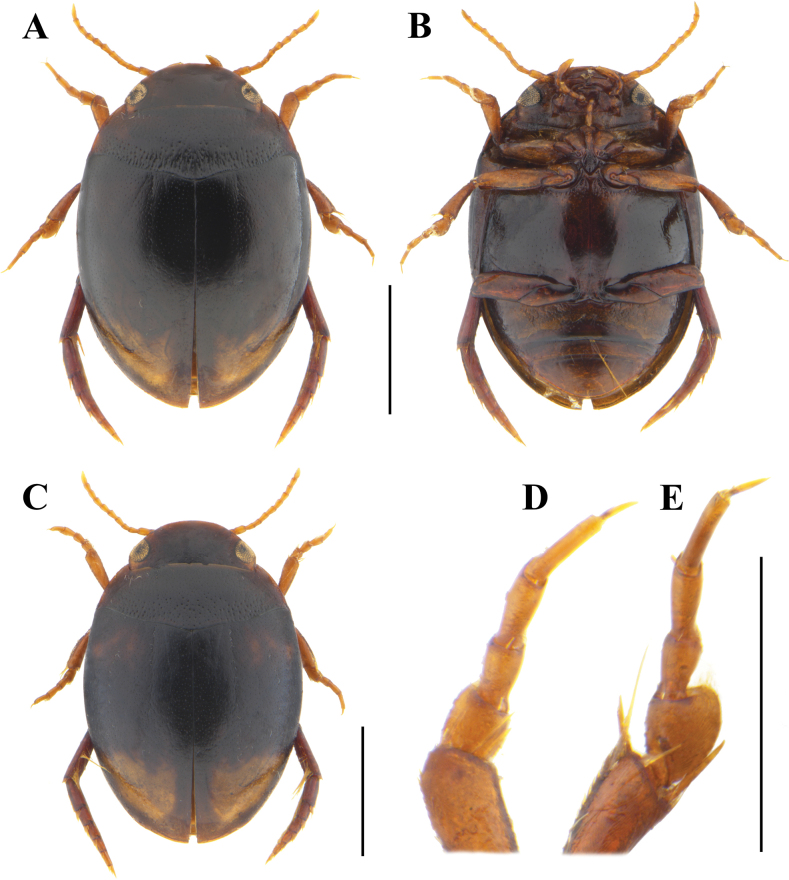
*Allopachrianigrocatta* sp. nov. (Maoershan, Guangxi) **A** habitus in dorsal view (holotype, male) **B** habitus in ventral view (holotype, male) **C** habitus in dorsal view (paratype, female) **D** male protarsus in dorsal view **E** male mesotarsus in dorsal view. Scale bars: 1.0 mm (**A–C**); 0.5 mm (**D, E**).

**Figure 2.**
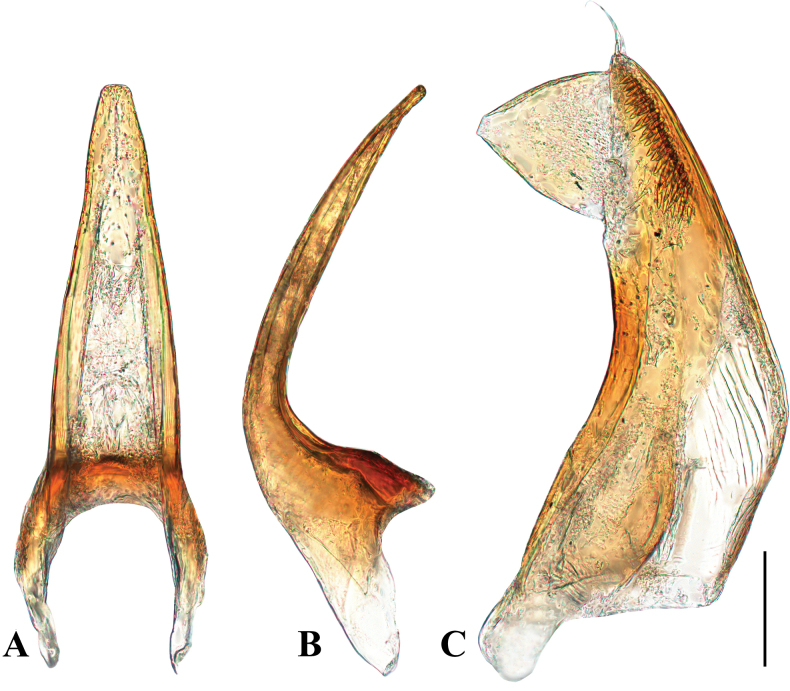
Male genitalia of *Allopachrianigrocatta* sp. nov. (holotype, male; Maoershan, Guangxi) **A** median lobe in ventral view **B** median lobe in lateral view **C** right paramere in lateral view. Scale bar: 0.1 mm.

Figs 1A–E , 2A–C , 4A , 6 , 7C

#### Type locality.

China, Guangxi Zhuang Autonomous Region, Guilin, Ziyuan County, Maoershan Mt., Huilong Temple, c. 25.9125°N, 110.4656°E; 1557.2 m.

#### Type material.

***Holotype*** • male (SYSU), labelled: “广西桂林市资源县 / 猫儿山迴龙寺 / 25.9125N，110.4656E / 1557.2m，30.viii.2020 / 姜卓寅 [p] // CHINA: Guangxi, Guilin / Ziyuan County, Maoershan Mt. / Huilong Temple / 25.9125N, 110.4656E / 1557.2m, 30.viii.2020 / Zhuoyin Jiang leg. [p] // HOLOTYPE / *ALLOPACHRIA* / *nigrocatta* sp. nov. / Jiang & Jia det. 2024 [red label, p]”. ***Paratype*** • 1 female (SYSU), same label data as holotype, with a paratype label, labelled: “PARATYPE / *ALLOPACHRIA* / *nigrocatta* sp. nov. / Jiang & Jia det. 2024 [red label, p]”.

#### Description of male holotype.

***Habitus*** (Fig. 1A) regularly oval, with continuous outline, broadest in 1/3 of elytral length, moderately convex. ***Colouration*.** Head dark brown to black, somewhat paler at clypeal margin, with small yellowish-brown area behind eyes; pronotum black with lateral margins reddish-brown; elytra black, apex with an irregularly shaped yellowish-brown spot not reaching suture; appendages yellowish-brown to reddish-brown; ventral side reddish-brown to black. ***Head*.** Moderately broad, c. 0.67 × width of pronotum, trapezoidal. Anterior margin of clypeus regularly rounded, without bead (Fig. 7C). Antenna with antennomeres long and slender. Shiny, microreticulation presents on anterior half of head and along eyes, consisting of well-impressed polygonal isodiametric meshes. Punctures spread sparsely and more distinct on vertex; setigerous punctures present along inner margin of eyes and anterolaterally to eyes in fronto-clypeal depressions. ***Pronotum*.** Strongly transverse (width/length ratio = 2.71), broadest between posterior angles. Lateral margins moderately curved, distinctly beaded. Shiny, microreticulation absent. Punctures and micropunctures spread sparsely and evenly; rows of setigerous punctures present along anterior margin; navel-like punctures present in posterior half. Posterior half also covered with some irregular longitudinal wrinkles. ***Elytra*.** Base as broad as pronotal base; lateral margins moderately curved. Shiny, microreticulation absent. Punctures and micropunctures spread sparsely and evenly; longitudinal rows of setigerous punctures incomplete. ***Legs*.** Protarsomere 1 minimally enlarged (Fig. 1D), mesotarsomere 1 distinctly enlarged (Fig. 1E), with adhesive setae on their ventral side; claws simple, metatarsal claws unequal. ***Ventral side*** (Fig. 1B). Prosternum sinuate anteriorly. Prosternal process heart-shaped, with distinct wide lateral beads in basal two thirds, apex obtuse; surface distinctly punctured. Metaventrite distinctly beaded on anterior margin; lateral parts of metaventrite (“metasternal wings”) narrow. Metacoxal lines prominent, divergent anteriorly. Metacoxal plates with some indistinct wrinkles. Metacoxal processes with a small triangular lobe, obscuring part of metatrochanter. Abdomen with five ventrites (III–IV fused). Microreticulation presents on abdominal ventrites, consisting of well-impressed polygonal isodiametric meshes. Punctures and micropunctures spread sparsely and evenly on metaventrite, metacoxae and abdominal ventrites; setigerous punctures present along anterior margin of metaventrite and medially on ventrites II–IV. ***Male genitalia*.** Median lobe of aedeagus gradually narrowing from base to apex in ventral view, apex truncate (Fig. 2A); ‘Lʼ-shaped in lateral view, moderately curved, apex obtuse (Fig. 2B). Parameres moderately broad, distal portion with a large triangular process on dorsal side, with a tuft of setae apically (Fig. 2C).

**Female** (Fig. 1C). Identical to male in habitus. Dorsal surface submatt, microreticulation presents on head, pronotum and elytra, more densely and longitudinally stretched on elytra. Pro- and mesotarsomeres not enlarged, without adhesive setae.

#### Variability.

Minor variability can be seen in dorsal colouration; there is an indistinct irregular reddish-brown spot on each elytral base in the paratype.

#### Measurements.

TL: 2.5–2.7 mm (mean value: 2.6 ± 0.1 mm); holotype: 2.7 mm. TL-h: 2.2–2.4 mm (mean value: 2.3 ± 0.1 mm); holotype: 2.4 mm. MW: 1.7–1.8 mm (mean value: 1.75 ± 0.05 mm); holotype: 1.8 mm.

#### Differential diagnosis.

*Allopachrianigrocatta* sp. nov. can be distinguished from other *Allopachria* species by its black colour pattern with a yellowish-brown spot on elytral apex. The shape of the median lobe and paramere is also unique. Female specimens can be easily identified by the submatt appearance.

#### Collection details.

On Maoershan Mt., *Allopachrianigrocatta* sp. nov. was collected syntopically with *Platambuspunctatipennis* Brancucci, 1984 from a pool with decaying leaves under a rock wall covered with flowing water (Fig. 4A).

#### Etymology.

The species name is a combination of “*nigro*-” from Latin “*nigriculus*” (= blackish) and “-*catta*” (= cat, in Chinese “mao” means cat), referring to its black appearance and the fact that it was collected from Maoershan Mt. The gender of the name is feminine.

#### Distribution.

So far, known only from the type locality on Maoershan Mt., northern Guangxi, China (Fig. 6).

### ﻿Additional records of Chinese *Allopachria*

#### ﻿ Allopachria bianae

Taxon classificationAnimaliaColeopteraDytiscidae

Wewalka, 2010

641B3278-7C26-5763-B214-B958B7090490

Figs 3A , 4B , 6

Allopachria bianae Wewalka, 2010: 31 (orig. descr.).

##### Material studied.

**China: Guangdong** • 11 spec. (SYSU, ZJCQ), Zhaoqing, Dinghu Distr., Dinghushan Mt., Qingyunsi management station, 23.1737°N, 112.5361°E, 241.2 m, 12.vii.2021, Z.Y. Jiang, Z.Q. Mai & Z.L. Liang leg. • 5 spec. (SYSU, ZJCQ), Zhaoqing, Dinghu Distr., Dinghushan Mt., Qingyunsi management station, 23.1732°N, 112.5354°E, 256.9 m, 17.x.2021, Z.Y. Jiang, Z.Q. Mai & W.C. Xie leg. • 5 spec. (SYSU, ZJCQ), Zhaoqing, Dinghu Distr., Dinghushan Mt., Dizhi management station, 23.1609°N, 112.5323°E, 88.7 m, 14.ix.2022, Z.Y. Jiang & W.C. Xie leg. • 18 spec. (SYSU, ZJCQ), Zhaoqing, Dinghu Distr., Dinghushan Mt., Qingyunsi management station, 23.1732°N, 112.5354°E, 256.9 m, 15.ix.2022, Z.Y. Jiang & W.C. Xie leg.

**Figure 3.**
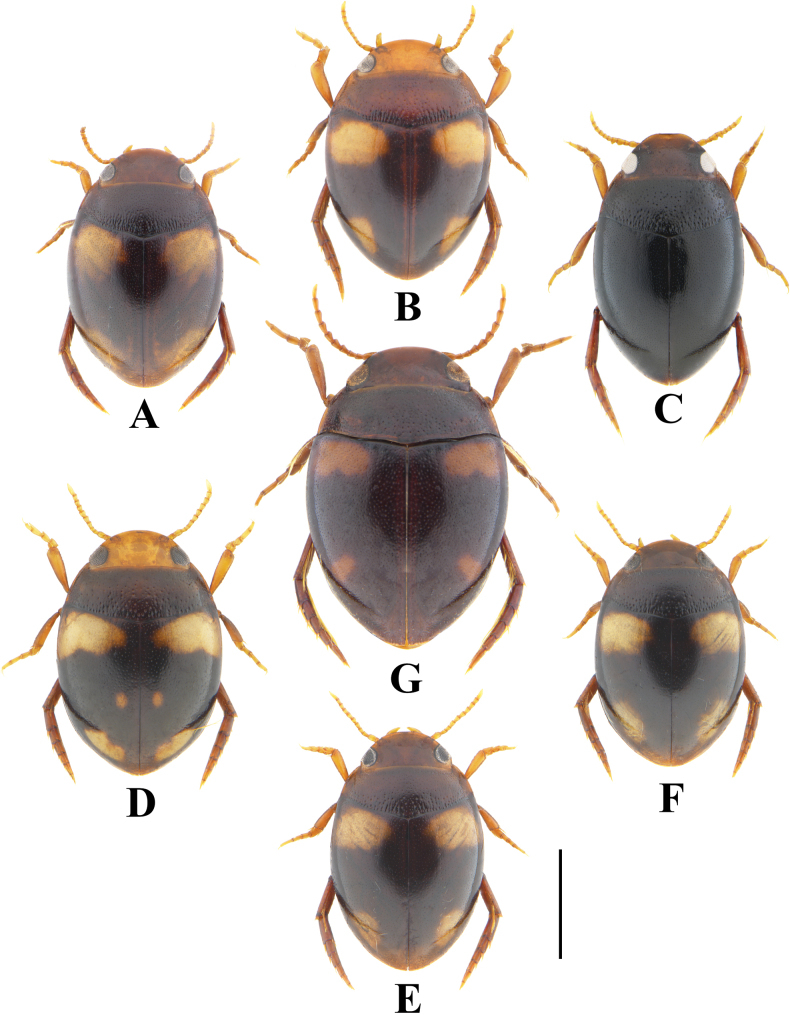
Habitus of *Allopachria* species **A***Allopachriabianae* (male; Dinghushan, Guangdong) **B***Allopachriadudgeoni* (male; Tianmushan, Zhejiang) **C***Allopachriajendeki* (male; Baihualing, Yunnan) **D***Allopachriamiaowangi* (male; Lingwei, Zhejiang) **E***Allopachriaschoenmanni* (male; Tianmushan, Zhejiang) **F***Allopachriaweinbergeri* (male; Lingwei, Zhejiang) **G***Allopachriayiae* (male; Machang Town, Guizhou). Scale bar: 1.0 mm.

##### Distribution.

So far known only from its type locality on Dinghushan Mt. (Guangdong) (Fig. 6).

##### Collection details.

**Figure 4.**
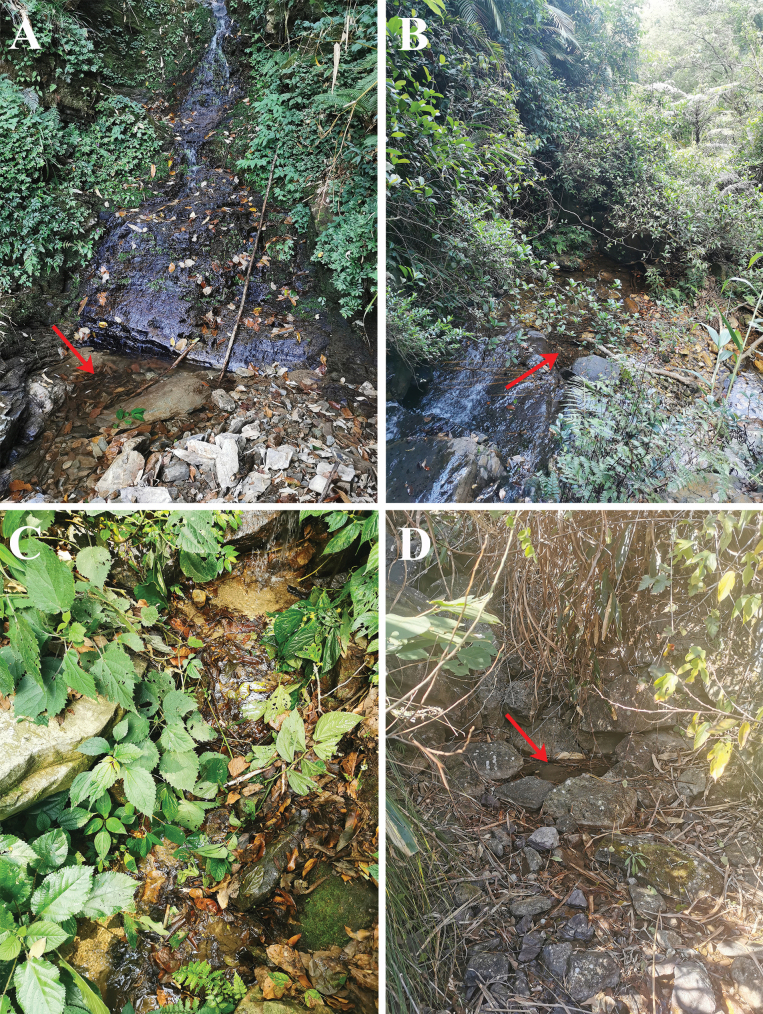
Habitat of *Allopachria* species **A** pool with decaying leaves under rock wall covered with flowing water, Maoershan, Guangxi (*A.nigrocatta* sp. nov.) **B** stream pool with decaying leaves, Dinghushan, Guangdong (*A.bianae*) **C** small stream with decaying leaves, Baihualing, Yunnan (*A.jendeki*) **D** small pool with flowing water surrounded by large stones, Tianmushan, Zhejiang (*A.schoenmanni*).

On Dinghushan Mt., *Allopachriabianae* was collected syntopically with *Neptosternuscoomani* Peschet, 1923 and *Neptosternuspunctatus* Zhao, Hájek, Jia & Pang, 2012 among decaying leaves in a stream pool (Fig. 4B).

#### ﻿ Allopachria dudgeoni

Taxon classificationAnimaliaColeopteraDytiscidae

Wewalka, 2000

4A7B9275-D637-5071-817C-5F964D92EB6D

Figs 3B , 6 , 7E

Allopachria dudgeoni Wewalka, 2000: 117 (orig. descr.); Wewalka 2010: 36 (fauna), Bian and Ji 2010: 64 (fauna), Bian and Ji 2012: 31 (fauna).

##### Material studied.

**China: Zhejiang** • 1 male (ZJCQ), Hangzhou, Lin’an Distr., Tianmushan Mt., Xiguan Stream S02, 30.3579°N, 119.4487°E, 609 m, x.2023, H.M. Zang & L.B. Han leg. • 1 female (ZJCQ), Hangzhou, Lin’an Distr., Tianmushan Mt., Waijunling, 30.3750°N, 119.4854°E, 537 m, xii.2023, H.M. Zang & L.B. Han leg.

##### Distribution.

Southeastern China (Guangdong, Guangxi, Hong Kong, Jiangxi, Zhejiang). First record from Zhejiang Province (Fig. 6).

#### ﻿ Allopachria jendeki

Taxon classificationAnimaliaColeopteraDytiscidae

Wewalka, 2000

B781ACE2-FC23-59F4-8758-5977415A33F7

Figs 3C , 4C , 6 , 7A

Allopachria jendeki Wewalka, 2000: 116 (orig. descr.); Wewalka 2010: 36 (fauna).

##### Material studied.

**China: Yunnan** • 1 male, 2 females (SYSU, ZJCQ), Baoshan, Baihualing Mt., Zaotang Stream, 25.3087°N, 98.7936°E, 1481.4 m, 11.v.2021, Z.Y. Jiang, Z.M. Yang, Z.Q. Mai & B.P. Huang leg.

##### Distribution.

Southwestern China (Yunnan) (Fig. 6).

##### Collection details.

The specimens were collected among decaying leaves and sand in a small stream on Baihualing Mt. (Fig. 4C).

#### ﻿ Allopachria miaowangi

Taxon classificationAnimaliaColeopteraDytiscidae

Wewalka, 2010

CC17C7B5-516A-5068-91CA-82E3C46D1DD3

**Figure 5.**
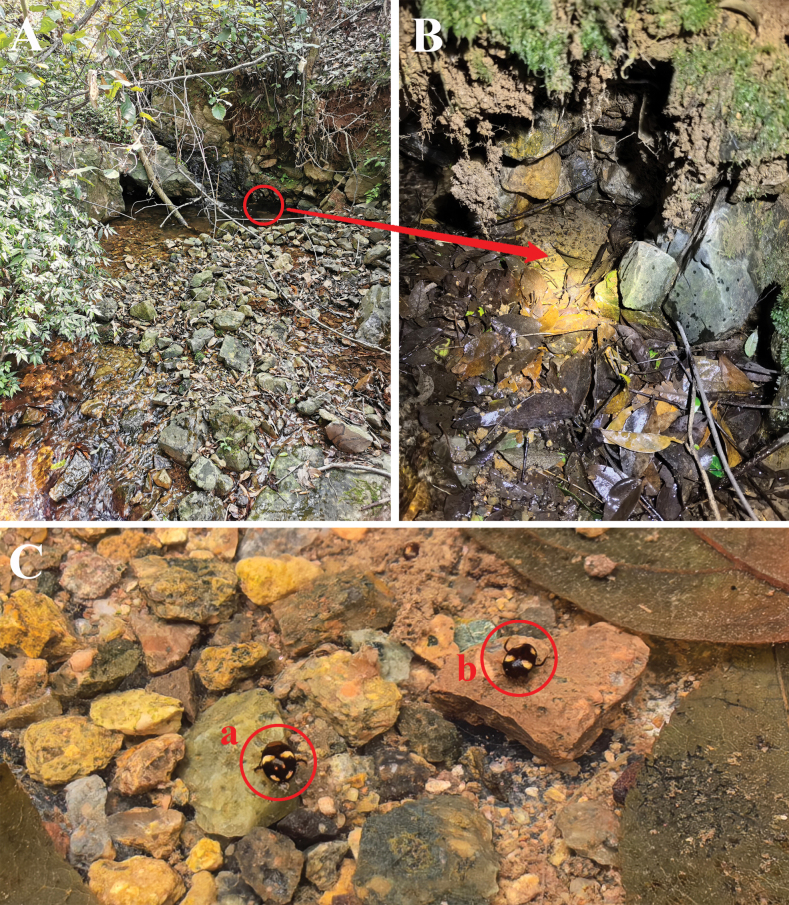
Habitat and live specimens of *Allopachriamiaowangi* and *Allopachriaweinbergeri***A** small stream under the forest (Lingwei, Zhejiang) **B** sand and stones by the margin of the stream (Lingwei, Zhejiang) **C** live specimens of *Allopachria* (**a***A.miaowangi***b***A.weinbergeri*).

Figs 3D , 5A, B, 5Ca, 6, 7F

Allopachria miaowangi Wewalka, 2010: 29 (orig. descr.); Bian and Ji 2012: 34 (fauna).
Allopachria dieterlei Wewalka, 2000: Bian and Ji 2010: 64 (misidentification).

##### Material studied.

**China: Zhejiang** • 4 males, 1 female (SYSU, ZJCQ), Quzhou, Kecheng Distr., Lingwei, 28.8507°N, 118.9372°E, 177.4 m, 2.v.2024, Z.Y. Jiang & Z.X. Mao leg. • 1 female (ZJCQ), Quzhou, Kecheng Distr., Lingwei, 28.8507°N, 118.9372°E, 177.4 m, 13.iv.2020, Z.Y. Jiang & Z.X. Mao leg.

##### Distribution.

**Figure 6.**
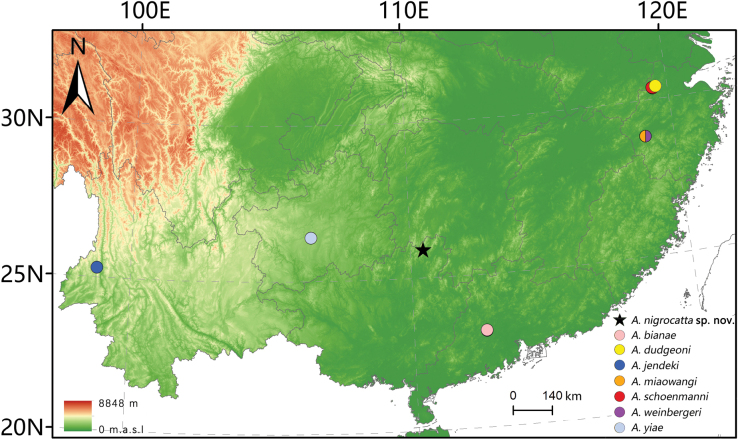
Map of distribution of *Allopachrianigrocatta* sp. nov. and additional records of other *Allopachria* species in China.

Eastern China (Hunan, Jiangxi, Zhejiang) (Fig. 6).

##### Collection details.

*Allopachriamiaowangi* was collected syntopically with *Allopachriaweinbergeri* under sand and stones by the margin of a small stream in Lingwei (Zhejiang) (Fig. 5A, B).

#### ﻿ Allopachria schoenmanni

Taxon classificationAnimaliaColeopteraDytiscidae

Wewalka, 2000

2ED97BB5-1C8D-5D39-82A2-53EF2A6BC253

Figs 3E , 4D , 6

Allopachria schoenmanni Wewalka, 2000: 113 (orig. descr.).

##### Material studied.

**China: Zhejiang** • 6 males, 2 females (SYSU, ZJCQ), Hangzhou, Lin’an Distr., Tianmushan Mt., Y437 roadside (Xiguan), 30.3483°N, 119.4509°E, 484.5 m, 20.xi.2023, Z.Y. Jiang leg. • 1 female (ZJCQ), Hangzhou, Lin’an Distr., Tianmushan Mt., Shuiduitang, 30.3682°N, 119.4255°E, 867 m, viii.2023, H.M. Zang & L.B. Han leg.

##### Distribution.

Eastern China (Anhui, Zhejiang) (Fig. 6).

##### Collection details.

Most specimens from Tianmushan Mt. were collected in a small pool with flowing water surrounded by large stones (Fig. 4D).

#### ﻿ Allopachria weinbergeri

Taxon classificationAnimaliaColeopteraDytiscidae

Wewalka, 2000

602DB28A-BE79-578A-8EA8-6BA5E0B1431D

Figs 3F , 5A, B, 5Cb, 6, 7G

Allopachria weinbergeri Wewalka, 2000: 112 (orig. descr.); Wewalka 2010: 36 (fauna).
Allopachria weinbergerorum Nilsson, 2007: 50 (as unjustified emendation of weinbergeri).

##### Material studied.

**China: Zhejiang** • 4 males, 2 females (SYSU, ZJCQ), Quzhou, Kecheng Distr., Lingwei, 28.8507°N, 118.9372°E, 177.4 m, 2.v.2024, Z.Y. Jiang & Z.X. Mao leg. • 1 female (ZJCQ), Quzhou, Kecheng Distr., Lingwei, 28.8507°N, 118.9372°E, 177.4 m, 13.iv.2020, Z.Y. Jiang & Z.X. Mao leg.

**Figure 7.**
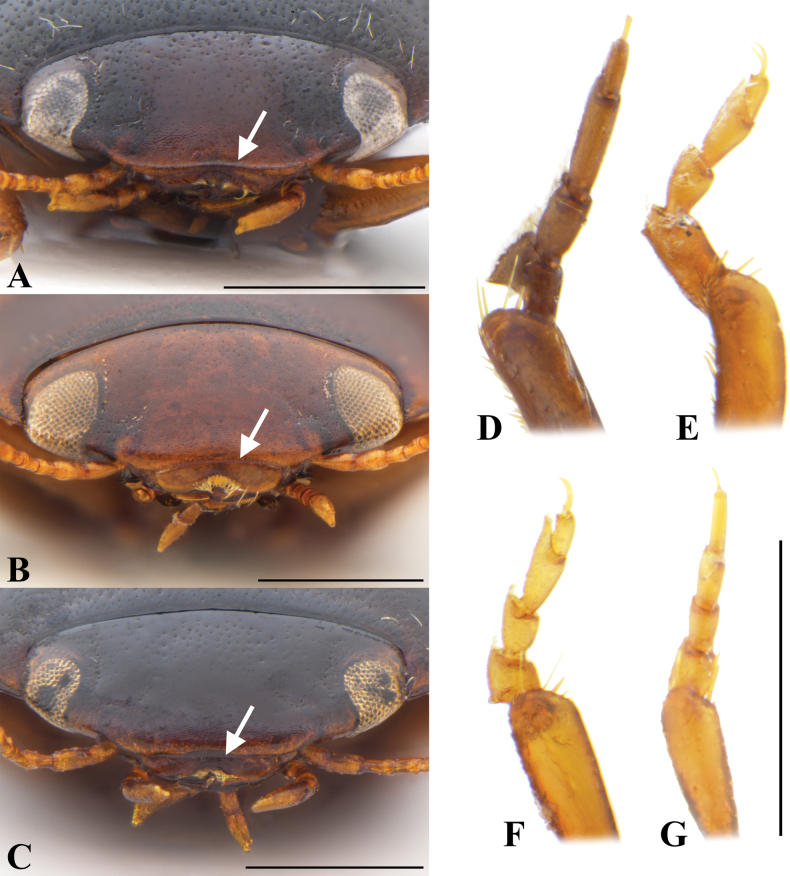
Morphological characters of *Allopachria* spp. **A–C** anterior aspect of head showing clypeus **D–G** male protarsus in dorsal view. **A***Allopachriajendeki***B, D***Allopachriayiae***C***Allopachrianigrocatta* sp. nov. **E***Allopachriadudgeoni***F***Allopachriamiaowangi***G***Allopachriaweinbergeri*. Scale bars: 0.5 mm.

##### Distribution.

Eastern China (Fujian, Guangdong, Zhejiang). First record from Zhejiang Province (Fig. 6).

##### Collection details.

*Allopachriaweinbergeri* was collected syntopically with *Allopachriamiaowangi* under sand and stones by the margin of a small stream in Lingwei (Zhejiang) (Fig. 5A, B).

#### ﻿ Allopachria yiae

Taxon classificationAnimaliaColeopteraDytiscidae

Bian, Guo & Ji, 2013

4285A226-5764-571C-9471-1BEBD087BFC7

Figs 3G , 6 , 7B , 7D

Allopachria yiae Bian, Guo & Ji, 2013: 85 (orig. descr.).

##### Material studied.

**China: Guizhou** • 3 males, 2 females (SYSU), Pingba, Machang Town, 13.viii.1982, Z.H. Huang leg.

##### Distribution.

Southwestern China (Chongqing, Guizhou). First record from Guizhou Province (Fig. 6).

### ﻿Key to Chinese *Allopachria* species (appropriate to males only)

**Table:** 

1	Anterior margin of clypeus with bead (Fig. 7A, B)	**2**
–	Anterior margin of clypeus without bead (Fig. 7C)	**10**
2	Anterior margin of clypeus with distinct bead (Fig. 7A)	**3**
–	Anterior margin of clypeus with very fine bead (Fig. 7B)	**7**
3	Elytra completely black, without spots (Fig. 3C)	***A.jendeki* Wewalka, 2000**
–	Elytra with two or three yellow to reddish-brown spots	**4**
4	Lateral margin of elytron with a longitudinal furrow	***A.manfredi* Wewalka, 2010**
–	Lateral margin of elytron without a longitudinal furrow	**5**
5	Male antennae modified, antennomeres 4 and 5 distinctly enlarged	***A.scholzi* Wewalka, 2000**
–	Male antennae not modified, with antennomeres long and slender	**6**
6	Size larger (TL: 2.1–2.2 mm); head yellow; elytra with three spots (see Wewalka 2000, fig. 25)	***A.ernsti* Wewalka, 2000**
–	Size smaller (TL: 1.7–1.8 mm); head black; elytra with two spots (see Wewalka 2000, fig. 8)	***A.taiwana* (Satô, 1990)**
7	Size larger (TL: 2.7–2.9 mm); male protarsomere 1 distinctly enlarged with indention on inner margin at basal half (Fig. 7D)	***A.yiae* Bian, Guo & Ji, 2013**
–	Size smaller (TL: 1.5–1.8 mm); male protarsomere 1 minimally enlarged	**8**
8	Male antennomere 5 distinctly enlarged, ‘L’-shaped (see Wewalka 2000, fig. 107	***A.froehlichi* Wewalka, 2000**
–	Male antennomere 5 moderately enlarged, with an obtuse tooth (see Wewalka 2000, fig. 108)	**9**
9	Median lobe of aedeagus in lateral view shorter and broader (see Wewalka 2000, fig. 44b)	***A.hautmanni* Wewalka, 2000**
–	Median lobe of aedeagus in lateral view longer and slender (see Wewalka 2000, fig. 43b)	***A.schillhammeri* Wewalka, 2000**
10	Size smaller (TL: 1.6 mm)	***A.jilanzhui* Wewalka, 2000**
–	Size larger (TL: ≥ 2.0 mm)	**11**
11	Apex of median lobe of aedeagus trifid	**12**
–	Apex of median lobe of aedeagus simple, not trifid	**16**
12	Elytra entirely microreticulate	**13**
–	Elytra without microreticulation	**14**
13	Punctation of pronotum regular in size, not navel-like; interstice of the tridentate apex of median lobe narrower (see Wewalka 2000, fig. 63a)	***A.wangi* Wewalka & Nilsson, 1994**
–	Punctation of pronotum irregular in size, partly coarse and navel-like; interstice of the tridentate apex of median lobe wider (see Wewalka 2000, fig. 64a)	***A.jaechi* Wewalka, 2000**
14	Male protarsomere 1 not modified, minimally enlarged	***A.flavomaculata* (Kamiya, 1838)**
–	Male protarsomere 1 modified, distinctly enlarged	**15**
15	Elytra with two or three spots (see Wewalka 2010, fig. 7a, b); middle part of the tridentate apex of median lobe longer than lateral parts (see Wewalka 2010, fig. 15a)	***A.komareki* Wewalka, 2010**
–	Elytra with two spots (see Bian and Ji 2010, fig. 1); middle part and lateral parts of the tridentate apex of median lobe equal in length (see Bian and Ji 2010, fig. 3)	***A.wuzhifengensis* Bian & Ji, 2010**
16	Male antennomeres 4 and 5 distinctly enlarged (see Wewalka 2000, fig. 113)	***A.friedrichi* Wewalka, 2000**
–	Male antenna not or minimally enlarged	**17**
17	Male protarsomere 1 modified, distinctly enlarged (Fig. 7E, F)	**18**
–	Male protarsomere 1 not modified, minimally enlarged (Fig. 7G)	**22**
18	Male protarsomere 1 elongate (Fig. 7E)	**19**
–	Male protarsomere 1 short (Fig. 7F)	**20**
19	Male protarsomere 1 about 1.5 times as long as protarsomere 2, insertion of protarsomere 2 at the apical part of protarsomere 1 (Fig. 7E)	***A.dudgeoni* Wewalka, 2000**
–	Male protarsomere 1 about 2 times as long as protarsomere 2, insertion of protarsomere 2 at the middle of protarsomere 1 (see Bian and Ji 2012, figs 15, 17)	***A.elongata* Bian & Ji, 2012**
20	Apex of median lobe of aedeagus pointed in ventral view (see Wewalka 2010, fig. 11a)	***A.miaowangi* Wewalka, 2010**
–	Apex of median lobe of aedeagus truncate in ventral view (see Wewalka 2010, fig. 12a)	**21**
21	Punctation of elytra stronger; median lobe of aedeagus in lateral view moderately curved (see Wewalka 2000, fig. 58b)	***A.dieterlei* Wewalka, 2000**
–	Punctation of elytra finer; median lobe of aedeagus in lateral view almost straight at middle part (see Wewalka 2010, fig. 12b)	***A.guangdongensis* Wewalka, 2010**
22	Elytra often completely black, sometimes with two reddish-brown spots (see Wewalka 2010, fig. 6a, b)	***A.hajeki* Wewalka, 2010**
–	Elytra with one or two yellowish-brown to reddish-brown spots	**23**
23	Size larger (TL: 2.5–2.7 mm); male mesotarsomere 1 distinctly enlarged (Fig. 1E); elytra often with one spot at apex (Fig. 1A)	***A.nigrocatta* sp. nov.**
–	Size smaller (TL: 2.0–2.4 mm); male mesotarsomere 1 not or minimally enlarged; elytra with two spots: one at base and one at apex	**24**
24	Apex of median lobe of aedeagus rounded or truncate in ventral view	**25**
–	Apex of median lobe of aedeagus pointed in ventral view	**27**
25	Apex of median lobe of aedeagus rounded in ventral view (see Wewalka 2000, fig. 51a)	***A.liselotteae* Wewalka, 2000**
–	Apex of median lobe of aedeagus truncate in ventral view	**26**
26	Median lobe of aedeagus in ventral view distinctly narrowed behind apical fourth (see Wewalka 2010, fig. 13a)	***A.bianae* Wewalka, 2010**
–	Median lobe of aedeagus in ventral view with sides almost parallel over entire length (see Ji et al. 2014, fig. 3)	***A.longyanensis* Ji, Guo & Bian, 2014**
27	Median lobe of aedeagus in ventral view broadened in middle, apex slender (see Bian and Ji 2012, fig. 10)	***A.yanfengi* Bian & Ji, 2012**
–	Median lobe of aedeagus in ventral view narrowed in middle, apex slightly broad	**28**
28	Median lobe of aedeagus in ventral view slightly narrowed in middle (see Wewalka 2000, fig. 50a)	***A.schoenmanni* Wewalka, 2000**
–	Median lobe of aedeagus in ventral view distinctly narrowed in middle	**29**
29	Median lobe of aedeagus in ventral view broader at apex (see Bian and Ji 2010, fig. 6)	***A.grandis* Bian & Ji, 2010**
–	Median lobe of aedeagus in ventral view narrower at apex (see Wewalka 2000, fig. 49a)	***A.weinbergeri* Wewalka, 2000**

### ﻿List of Chinese *Allopachria* Zimmermann species, including their distribution

*Allopachriabianae* Wewalka, 2010 Guangdong

*Allopachriadieterlei* Wewalka, 2000 Hunan

*Allopachriadudgeoni* Wewalka, 2000 Guangdong, Guangxi, Hong Kong, Jiangxi, Zhejiang

*Allopachriaelongata* Bian & Ji, 2012 Guangxi

*Allopachriaernsti* Wewalka, 2000 Guangxi; North Vietnam

*Allopachriaflavomaculata* (Kamiya, 1838) Guangxi; Japan

*Allopachriafriedrichi* Wewalka, 2000 Hunan

*Allopachriafroehlichi* Wewalka, 2000 Hong Kong

*Allopachriagrandis* Bian & Ji, 2010 Jiangxi

*Allopachriaguangdongensis* Wewalka, 2010 Guangdong, Guangxi

*Allopachriahajeki* Wewalka, 2010 Yunnan

*Allopachriahautmanni* Wewalka, 2000 Anhui

*Allopachriajaechi* Wewalka, 2000 Hainan

*Allopachriajendeki* Wewalka, 2000 Yunnan

*Allopachriajilanzhui* Wewalka, 2000 Guangxi, Hunan

*Allopachriakomareki* Wewalka, 2010 Guangdong

*Allopachrialiselotteae* Wewalka, 2000 Guangxi

*Allopachrialongyanensis* Ji, Guo & Bian, 2014 Fujian

*Allopachriamanfredi* Wewalka, 2010 Guangdong

*Allopachriamiaowangi* Wewalka, 2010 Hunan, Jiangxi, Zhejiang

*Allopachrianigrocatta* sp. nov. Guangxi

*Allopachriaschillhammeri* Wewalka, 2000 Hunan

*Allopachriaschoenmanni* Wewalka, 2000 Anhui, Zhejiang

*Allopachriascholzi* Wewalka, 2000 Yunnan

*Allopachriataiwana* (Satô, 1990) Taiwan

*Allopachriawangi* Wewalka & Nilsson, 1994 Taiwan

*Allopachriaweinbergeri* Wewalka, 2000 Fujian, Guangdong, Zhejiang

*Allopachriawuzhifengensis* Bian & Ji, 2010 Hunan, Jiangxi

*Allopachriayanfengi* Bian & Ji, 2012 Guangxi

*Allopachriayiae* Bian, Guo & Ji, 2013 Chongqing, Guizhou

## Supplementary Material

XML Treatment for Allopachria nigrocatta

XML Treatment for Allopachria bianae

XML Treatment for Allopachria dudgeoni

XML Treatment for Allopachria jendeki

XML Treatment for Allopachria miaowangi

XML Treatment for Allopachria schoenmanni

XML Treatment for Allopachria weinbergeri

XML Treatment for Allopachria yiae

## ﻿Additional information

### Author ORCIDs

Zhuo-yin Jiang https://orcid.org/0000-0001-6825-4283

Feng-long Jia https://orcid.org/0000-0003-2391-5038

Bei-xin Wang https://orcid.org/0000-0002-5253-8799

### Data availability

All of the data that support the findings of this study are available in the main text.
